# Pathogenic HNF1A Variant in an Indonesian Family: Atypical Management of MODY3 Guided by Patient Comorbidity

**DOI:** 10.1016/j.aed.2025.10.006

**Published:** 2025-10-16

**Authors:** Ardy Wildan, Fergie Marie Joe Grizella Runtu, Mentari Kasih, Selvi Nafisa Shahab, Dicky Levenus Tahapary

**Affiliations:** 1 Division of Endocrinology, Metabolism, and Diabetes, Department of Internal Medicine, Faculty of Medicine Universitas Indonesia, Cipto Mangunkusumo Hospital, Jakarta, Indonesia; 2 Clinical Research Unit, Cipto Mangunkusumo Hospital, Jakarta, Indonesia; 3 Metabolic, Cardiovascular, Aging Cluster, Indonesia Medical Research Insitute, Jakarta, Indonesia

**Keywords:** DPP-4 inhibitor, HNF1A, MODY, next-generation sequencing

## Abstract

**Background/Objective:**

Maturity-onset diabetes of the young (MODY) is an autosomal dominant form of monogenic diabetes frequently misdiagnosed as type 1 or type 2 diabetes. Identifying the specific subtype is crucial, as several subtypes, such as *HNF1A*-MODY (MODY-3), are typically well-controlled with sulfonylureas.

**Case Report:**

A 19-year-old male with a history of diabetes presented with right-sided weakness, aphasia, and facial asymmetry. He was admitted for gamma knife radiosurgery to treat a left basal ganglia arteriovenous malformation. One month prior to this admission, he had undergone surgical evacuation of an intracranial hemorrhage—a complication of his arteriovenous malformation—at another hospital. He was discharged on a basal-bolus insulin regimen due to hyperglycemia during that hospitalization and was subsequently referred to our center. During the current admission, glycemic control was achieved, allowing gradual reduction of insulin dose and transition to oral sitagliptin/metformin XR upon discharge. Genetic testing later confirmed a pathogenic c.160C>T variant in the *HNF1A* gene, which was also identified in his mother and younger sibling. At the 2-month follow-up, due to sustained glycemic control, his treatment was simplified to sitagliptin 100 mg monotherapy.

**Discussion:**

Although sulfonylureas are typically the first-line treatment for *HNF1A*-MODY, individualized therapy was required due to the patient's neurological comorbidities. dipeptidyl peptidase-4 inhibitor therapy provided effective glycemic control and offered potential neurocognitive benefits.

**Conclusion:**

This case underscores that while genetic confirmation of *HNF1A*-MODY guides therapy, treatment should be individualized based on comorbidities and prior medication history to optimize glycemic control.

**Highlights**

• First confirmed MODY case in Indonesia using in-house targeted next-generation sequencing
• Pathogenic HNF1A variant (MODY3) identified via local genetic testing
• While sulfonylureas are the standard first-line therapy for HNF1A-MODY, this patient was managed with a dipeptidyl peptidase-4 inhibitor due to cognitive impairment, which made him vulnerable to hypoglycemia
• Demonstrates feasibility of targeted genetic diagnostics in developing countries

**Clinical Relevance**

This case report showcases the implementation of in-house targeted genetic testing for MODY in Indonesia. This advancement enables accurate diagnosis, which is crucial for appropriate treatment and family screening, ultimately improving diabetes care precision medicine in a resource-limited setting.

## Background

Maturity-onset diabetes of the young (MODY), the most common form of monogenic diabetes, accounts for up to 5% of all diabetes cases. It is frequently misdiagnosed as type 1 or type 2 diabetes due to overlapping clinical features.^1^ Genomic sequencing, essential for definitive diagnosis, is largely unavailable in low-resource settings such as Indonesia. Consequently, MODY is often managed inappropriately as type 1 or type 2 diabetes. Inadequate access to autoantibody and C-peptide testing further impedes adherence to diagnostic guidelines. This misclassification hinders the application of precision treatment, such as using sulfonylureas in hepatocyte nuclear factor 1 alpha (*HNF1A*) MODY (previously known as MODY3), leading to suboptimal treatment outcomes, increased risk of complications, and elevated long-term health care costs.^2^

In response to these challenges, the Indonesian government has recently launched a national genomic program aimed at advancing precision medicine. In alignment with this initiative, we established the GENESIS-ID study (Genomic Exploration of Young-onset Diabetes in Indonesia), a multicenter prospective cohort designed to develop a clinical-genomic diabetes registry and a targeted MODY diagnostic panel. Here, we report the first confirmed case of MODY in Indonesia diagnosed using in-house targeted next-generation sequencing (tNGS) using a gene panel covering 5 common MODY genes: *GCK, HNF1A, HNF4A, INS,* and *KCNJ11*. While limited, it includes clinically actionable subtypes. Although this targeted approach has limitations, the broader GENESIS-ID project incorporates Ministry of Health-supported whole-genome sequencing to facilitate comprehensive MODY subtyping and the discovery of novel variants. This case represents a pivotal step toward integrating genomic diagnostics into routine diabetes care in Indonesia.

## Case Description

We received a referral for a 19-year-old male inpatient with diabetes scheduled for gamma knife radiosurgery following the diagnosis of a left basal ganglia arteriovenous malformation by computed tomography angiography and digital subtraction angiography.

One month prior to presentation, the patient experienced a loss of consciousness. An emergency computed tomography scan revealed an intracranial hemorrhage, which was managed with surgical hematoma evacuation and a decompressive craniectomy at another hospital. He was hospitalized for 3 weeks, during which he developed pneumonia and hyperglycemia requiring insulin therapy. Postoperatively, he exhibited a left central facial palsy, dysarthria, aphasia, and right hemiplegia. Upon discharge, he was prescribed insulin detemir 22U daily and insulin aspart 12U three times a day. He was subsequently referred to our hospital for further care.

### History of Past Illness

The patient had been diagnosed with diabetes 2 years prior and had been inconsistently taking glimepiride with a history of repeated hypoglycemia. His most recent glycated hemoglobin (HbA1c) was 7%.

### Personal and Family History

**Fig. 1:**
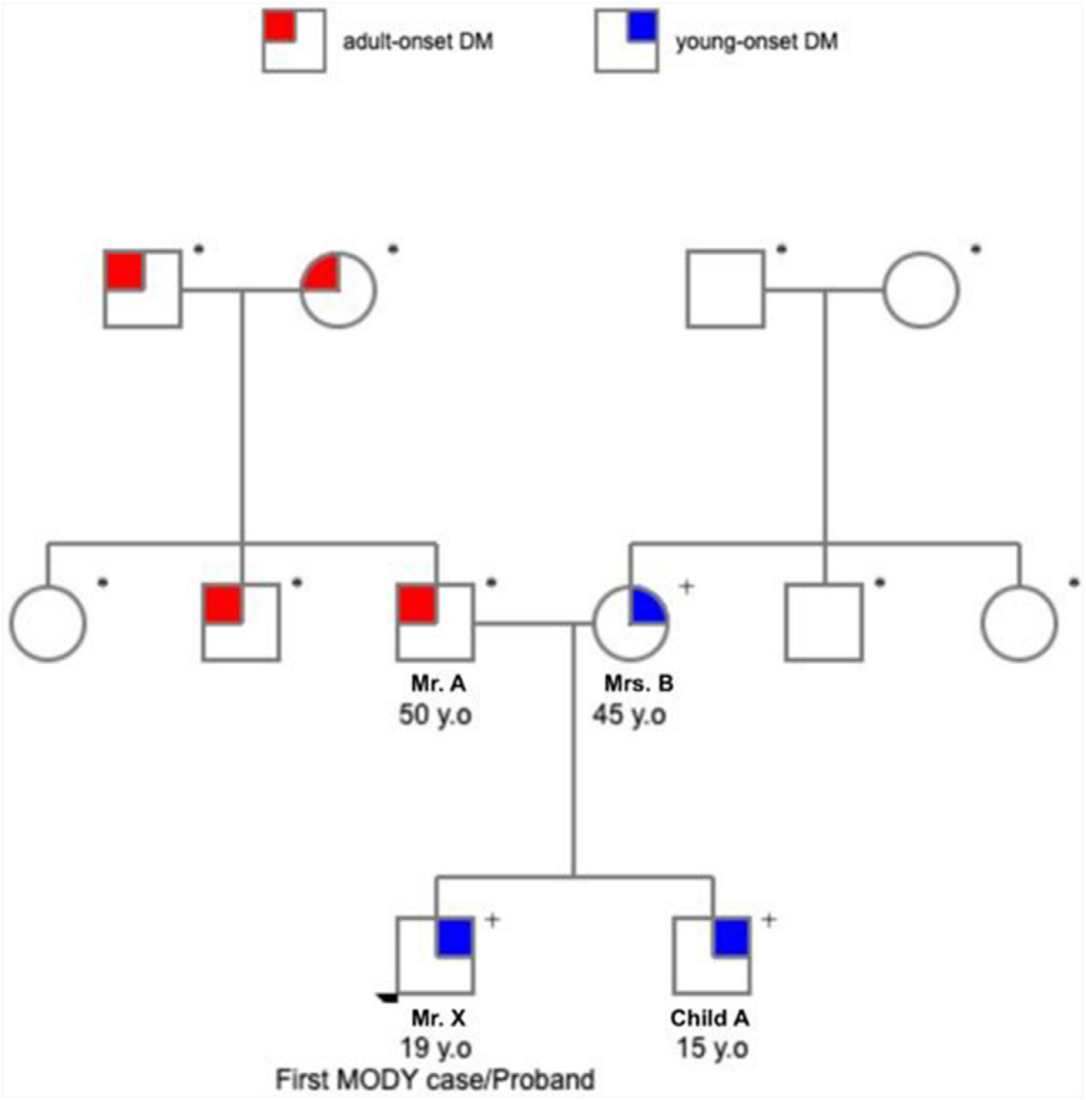
Pedigree of the patient’s family showing an autosomal dominant inheritance pattern of the pathogenic MODY3 variant in *HNF1A*. ∗: unknown status of the variant in the family tree due to genetic testing not yet performed; +: confirmed pathogenic mutation of *HNF1A.DM* = diabetes mellitus.

A multigenerational history of diabetes, consistent with an autosomal dominant inheritance pattern, was observed (Fig. 1). His mother was diagnosed with diabetes at 26 years old, during her first pregnancy. She was managed with glimepiride 2 mg; her last recorded HbA1c was 8.8%. None of her siblings were reported to be having diabetes. The patient’s younger sibling was diagnosed with diabetes during junior high. His father and paternal grandparents were diagnosed with diabetes later in life.

### Physical and Laboratory Findings

The patient’s vital signs were within normal limits. His body mass index was 18.9 kg/m^2^. General examination findings were unremarkable. Neurological examinations revealed expressive aphasia and right hemiparesis.

Laboratory investigations showed a normal complete blood count. HbA1c was well controlled at 5.5%. The lipid profile was low-density lipoprotein 96 mg/dL, high-density lipoprotein 45 mg/dL, and triglycerides 104 mg/dL.

### Clinical Course and Management

The patient was diagnosed with mixed transcortical aphasia, right-sided cranial nerve VII dan XII paresis, right hemiparesis, and a left basal ganglia arteriovenous malformation with a history of rupture. Based on the clinical features, MODY Exeter calculator determined the likelihood of 75.5% for MODY.^3^ Given the logistical and financial constraints associated with antibody testing at our center, targeted genetic testing was prioritized.

**Fig. 2:**
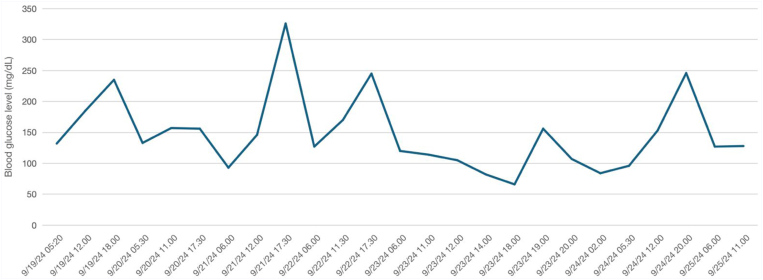
Patient’s glucose profile during admission at our hospital.

During hospitalization, insulin requirements gradually decreased (Fig. 2). He was discharged on sitagliptin/metformin XR pending further evaluation. A tNGS test (GridION platform, Oxford Nanopore Technologies) was initiated using a MODY gene panel (*HNF1A, HNF4A, GCK, KCNJ11*, and *INS*).

The HbA1c increased from 5.5% to 7.2% within 2 months after discontinuing insulin. While this increase indicates deteriorating glycemic control, it remained within an acceptable range given the clinical priority of avoiding hypoglycemia during neurological recovery. Fasting C-peptide level was measured at 1.1 ng/mL (*N*: 0.5-2 0.0 ng/mL), making T1D less likely. Homeostatic model assessment of insulin resistance was calculated at 1.3 (normal <1.0), supporting MODY or early-onset type 2 diabetes as a differential diagnosis.

**Fig. 3:**
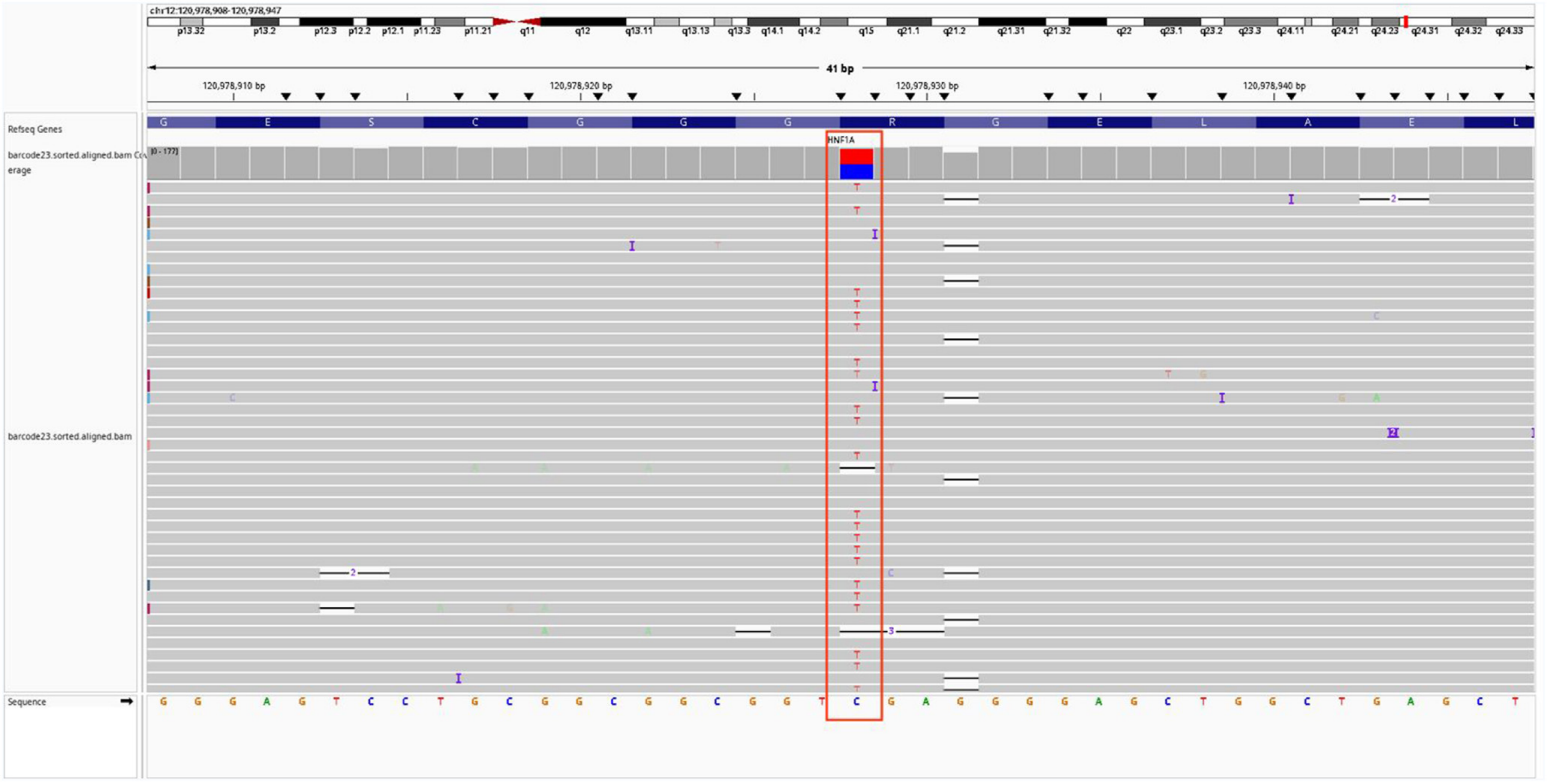
Alignment of *HNF1A* variant c.160C>T in the 19-year-old patient. The red box indicates the variant site.

The genetic analysis revealed a pathogenic variant, c.160C>T in the *HNF1A* gene (Fig. 3), confirming *HNF1A*-MODY. Subsequent family testing confirmed the same variant in the patient’s mother and brother. His father declined testing (Fig. 1). In the subsequent follow-up period, the patient reported no history of hypoglycemia while on sitagliptin/metformin XR. Given his cognitive limitations and hypoglycemia risk, treatment was simplified to sitagliptin 100 mg monotherapy. However, at 6 months, HbA1c rose to 8.8% due to poor adherence to therapy. As his cognitive function had improved considerably after neurorestoration, we transitioned him to gliclazide MR 60 mg, aligning with standard *HNF1A*-MODY treatment guidelines.

## Discussion

This case demonstrates the utility of tNGS in diagnosing MODY in Indonesia. While Broome’s diagnostic algorithm recommends testing for islet-cell autoantibodies (GADA, IA-2A, and ZnT8) before genetic testing,^4^ these tests were unavailable at our center and would require costly third-party laboratory services. Since the tNGS for MODY genes was available at our center at no additional cost, it became the practical choice.

Genetic analysis identified a pathogenic c.160C>T mutation in the HNF1A gene, located on chromosome 12q24, predicted to introduce a premature stop codon, resulting in protein truncation and functional loss. This confirmed a diagnosis of *HNF1A*-MODY subtype, which is reported to be the most prevalent MODY type, accounting for 30% to 50% of all MODY cases. Interestingly, although the mutation was also present in the patient’s mother, whose parents reportedly did not have diabetes, the possibility of a de novo mutation or incomplete penetrance remains.

While *HNF1A*-MODY is the most common form in Caucasian populations, its prevalence varies across Asia. This was demonstrated in a study by Xu et al of 146 suspected MODY cases in the Chinese population, which found no instances of *HNF1A*-MODY after screening for common subtypes.^5^ However, despite limited data from Southeast Asia, *HNF1A*-MODY had been identified in the region. Plengvidhya et al^6^ identified 3 unrelated MODY3 cases with distinct *HNF1A* variants in Thailand. Ang et al in Singapore also reported a 17.5% diagnostic rate of MODY among 175 suspected cases referred to a regional monogenic diabetes center by endocrinologists, with *HNF1A* being the most commonly mutated gene.^7^ These regional variations suggest even differing frequencies of pathogenic variants among Asians.

The pathophysiology of *HNF1A*-MODY directly informs treatment. The core defect is an impaired glucose-stimulated insulin secretion, making patients particularly sensitive to sulfonylureas, the recommended first-line therapy.^8^ At discharge, while waiting for genetic confirmation, the patient received sitagliptin/metformin XR rather than glimepiride due to a history of hypoglycemic episodes. When the diagnosis of *HNF1A*-MODY was confirmed, he had achieved reasonable glycemic control (fasting plasma glucose 130 mg/dL, HbA1c 7.2%) and reported no episodes of hypoglycemia. Although sulfonylureas are recommended, *HNF1A*-MODY patients were found to be 4 times more responsive to sulfonylurea compared to patients with type 2 diabetes melitus, therefore imposing a greater risk of hypoglycemia. Given our patient’s cognitive impairment and vulnerability to hypoglycemia, treatment was adjusted to monotherapy with sitagliptin 100 mg after a family discussion. The lack of home glucose monitoring capability, due to the patient’s needle phobia combined with his cognitive limitation, limited our ability to titrate more aggressively, as we prioritized safety over optimal glycemic targets.

Evidence of the use of dipeptidyl peptidase-4 (DPP-4) inhibitors in MODY is limited. While its role as monotherapy remains understudied, several reports support its efficacy when used as an adjunctive agent with sulfonylureas.^9^^,^^10^ Østoft et al found impaired incretin effect and glucose intolerance in patients with HNF1A-MODY following oral glucose intake.^11^ This may explain why DPP-4 inhibition effectively controlled this patient’s blood glucose levels by improving the deficient incretin response. However, this study did not specify the antidiabetic medication used by subjects nor evaluate the effect of the medications on plasma glucose control.^11^

Additionally, dysglycemia has been linked to cognitive impairment, with some antidiabetic drugs showing protective effects. DPP-4 inhibitors, along with glucagon-like peptide-1 receptor agonists, sodium-glucose cotransporter-2 inhibitors, and metformin, have been associated with cognitive benefits. Meanwhile, insulin and sulfonylureas may have adverse cognitive outcomes, primarily due to hypoglycemia-associated neurocognitive dysfunction.^12^ Meng et al reviewed 6 clinical trials evaluating the impact of DPP-4 inhibitors on cognitive outcomes and found a general increase in Mini-Mental State Examination or Instrumental Activities of Daily Living in subjects with DPP-4 inhibitors.^13^

In our patient, this individualized approach prioritized safety over optimal glycemic targets during the acute recovery phase. While HbA1c worsened from 5.5% to 8.8% on sitagliptin therapy, this strategy avoided hypoglycemia during a period when the patient had severe cognitive impairment limiting his ability to recognize and respond to hypoglycemic symptoms. However, as his neurological function improved and glycemic control became suboptimal, we transitioned to gliclazide MR as the standard first-line therapy for *HNF1A*-MODY. This case illustrates that while sulfonylureas remain the gold standard for *HNF1A*-MODY, temporary use of alternative agents may be appropriate when patient-specific factors warrant a modified approach.

## Conclusion

This case highlights the atypical management of a patient diagnosed via tNGS with HNF1A-MODY, in which cognitive impairment and a history of hypoglycemia guided the selection of a DPP-4 inhibitor over the conventional use of sulfonylureas. Although deviating from standard guidelines, this individualized approach may reduce short-term complications such as hypoglycemia and mitigate potential long-term issues, including cognitive dysfunction.

## Statement of Patient Consent

Informed consent from the patient was obtained for this case report.

## Disclosure

The authors have no conflicts of interest to disclose.
